# Mesenchymal stem cell use in acute respiratory distress syndrome: a potential therapeutic application

**DOI:** 10.2144/fsoa-2020-0048

**Published:** 2020-05-12

**Authors:** Julien Freitag, James Wickham, Kiran Shah, Abi Tenen

**Affiliations:** 1 Charles Sturt University, School of Biomedical Sciences, Orange, NSW, Australia; 2 Magellan Stem Cells, Box Hill, Victoria, Australia; 3 Melbourne Stem Cell Center, Box Hill, Victoria, Australia; 4 Monash University, School of Primary Healthcare, Faculty of Medicine, Clayton, Victoria, Australia

## Abstract

Acute respiratory distress syndrome (ARDS) is a condition of acute respiratory failure resulting from noncardiogenic pulmonary edema. It may occur as a consequence of lung infection, sepsis, trauma, aspiration or drug reaction. The pathogenesis of ARDS is understood to be an unregulated inflammatory cascade with both endothelial and epithelial layer damage leading to alveolar fluid collection and pulmonary edema. Despite improved understanding of the cause of ARDS, treatment remains supportive with a mortality rate ranging from 25–40%. Preclinical and early phase clinical trials have highlighted the potential role of mesenchymal stem cells in combating the inflammatory cascade through immunomodulatory mechanisms and assisting in tissue repair.

## Lay abstract

Acute respiratory distress syndrome (ARDS) is a condition of sudden respiratory failure due to fluid in the lungs as a consequence of factors such as trauma and bacterial or viral lung infections. ARDS is understood to occur as a result of uncontrolled inflammation. Conventional treatment is supportive only with ARDS having a high death rate of up to 40%. Evidence from both preclinical and clinical trials highlight the potential role of mesenchymal stem cells in combating the inflammatory reactions that cause ARDS as this therapy both modifies the immune response and provides assistance with tissue repair.

Acute respiratory distress syndrome (ARDS) is a syndrome of acute respiratory failure resulting from acute and diffuse inflammatory lung injury leading to noncardiogenic pulmonary edema. Its development is commonly associated with bacterial and viral pneumonia though may also be a consequence of sepsis, severe trauma, aspiration and drug reactions [1,2]. Mortality of ARDS ranges from 25 to 40% with treatment primarily involving supportive care [3,4].

## Pathogenesis

The pathogenesis of ARDS is characterized by an unregulated inflammatory cascade with increased lung endothelial and epithelial permeability. Cell injury-associated endogenous molecules and microbial products are seen to bind to receptors on both epithelial cells and alveolar macrophages activating an immune response. Resultant unrestrained generation of reactive oxygen species, leukocyte proteases, chemokines and inflammatory cytokines results in progressive lung injury. This immune mediated response has been denoted as a ‘cytokine storm’ [5]. In effect it is the unregulated and excessive immune reaction that contributes to alveolar injury leading to the resultant serious and life-threatening clinical complications of ARDS [6,^7^].

In addition to the unrestrained excessive inflammation associated with ARDS, it is an observed endothelial and epithelial layer damage and a resultant increased cell lining permeability that results in alveolar fluid collection and pulmonary edema. Disruption of the endothelial cell layer, in particular the vascular endothelial cadherin bonds, leads to the leakage of water, solutes, leukocytes, platelets and inflammatory molecules into the alveolar space with reduction/failure of alveolar fluid clearance and subsequent pulmonary edema [8].

Importantly, it appears environmental factors also contribute to a risk of ARDS development and the severity of complications. Active and passive cigarette smoking, chronic alcohol use and obesity have been found to be associated with both development of ARDS and a poor outcome [9–12].

The successful treatment and eventual resolution of ARDS requires repair of endothelial and epithelial barriers with resultant effective alveolar fluid clearance and removal of inflammatory cells and cytokines from air spaces and lung parenchyma.

## Potential role of mesenchymal stem cells

Both preclinical trials and early Phase I/II clinical trials have supported the potential benefit of mesenchymal stem cell (MSC) therapy in the treatment of ARDS. MSCs are a heterogenous cell population first characterized by Dr Alexander Friedenstein and recognized to display plasticity and multipotency. They are found in numerous tissues including bone marrow, adipose tissue, skeletal muscle and peripheral and cord blood.

While initial focus on MSC therapy has been on their ability to differentiate along a mesodermal lineage it is now understood that their primary mechanism of action is through both paracrine mechanisms and cell–cell interaction [13]. MSCs are observed to suppress inflammatory T-cell proliferation and inhibit maturation of monocytes and myeloid dendritic cells resulting in an immunomodulatory and anti-inflammatory effect. Along with their immunomodulatory and differentiation potential, MSCs have been shown to express essential anti-inflammatory cytokines such as IL-1RA, IL-8 and IL-10 and an array of bioactive molecules that stimulate local tissue repair [14–16].

MSCs for allogeneic application are able to be sourced from various donor tissues. While bone marrow has commonly been used as a source of MSCs it has a relative paucity of MSCs with the MSC population comprising only 0.001–0.02% of the mononucleated cells isolated from bone marrow aspirate [17,18]. In contrast, human adipose tissue yields MSC numbers of 1–10% of the nucleated cell population [19]. In addition, adipose derived MSCs have been shown to retain their stemness (i.e. ability to self renew and differentiate), multipotency and their inherent immunomodulatory properties following long-term passaging making them an attractive option for allogeneic use [20–23]. Similarly, human umbilical cord perivascular cells are another source of MSCs and express significant promise with retention of MSC properties over extended *in vitro* expansion/culture [24].

## Safety of MSC therapy

Meta-analysis of trials involving a total of over 1000 participants receiving intravascular MSC therapy for various clinical conditions including ischemic heart disease, graft *versus* host disease, ischemic stroke, Crohn’s disease and cardiomyopathy, with follow-up up to 92 months, has indicated no significant adverse events other than transient fever [25]. No serious adverse events including death or malignancy were observed.

## Preclinical research

Preclinical research in animals with induced lung injury have indicated the potential of MSC therapy to reduce mortality, reduce inflammation and limit lung injury [26,27]. Ortiz and colleagues described the inhibition of bleomycin induced inflammation and fibrosis in the lungs of mice through the application of MSCs [26]. They noted that the bleomycin induced increase in TNF-α and IL-1 to be inhibited by the expression of MSC derived IL-1RA and the additional inhibition of TNF-α production by activated macrophages. Using intrapulmonary delivery of MSCs in a mice model, Gupta and colleagues were able to show effective attenuation of endotoxin induced acute lung injury [27]. A reduction in broncho-alveolar lavage protein (a measure of endothelial and epithelial permeability) and excess lung water (a measure of pulmonary edema) in MSC treated mice indicated effective reparative benefit following MSC application. In addition reduced expression of inflammatory cytokines and increased expression of anti-inflammatory cytokines in both broncho-alveolar washings and blood plasma samples were observed.

In a preclinical *ex vivo* analysis of human lung parenchyma exposed to high-dose intra-alveolar endotoxin, allogeneic human MSC therapy has been shown to markedly reduce lung edema and restore native lung endothelial and epithelial permeability [28]. The same trial noted similar benefit with the use of the MSC conditioned media (CM) suggesting a paracrine effect through both cytokines and exosomal/extracellular vesicle (EV) expression. The release of KGF by MSCs (and also found in CM) has been postulated to play a significant role in repair and injury reduction.

MSCs have further been seen to transfer mitochondria to injured alveolar epithelium with observed restoration of normal ATP levels and subsequent reduction in protein leak and improvement of surfactant secretion [29]. Transfer was observed by cell–cell interaction with MSCs directly attaching to alveolar epithelium by forming gap junctional channels with the release of mitochondria containing microvesicles that were engulfed by the epithelial cells.

In addition to these mechanisms, MSCs have been noted to have an antimicrobial role and thus, perhaps having benefit in combating primary or secondary lung infections [30]. Preclinical trials have shown MSCs to release LL-37, an antimicrobial peptide, which enhances monocyte microbial phagocytosis, thus exerting an antimicrobial effect and stimulating bacterial clearance [31,32].

In a xenograft preclinical trial assessing the safety and efficacy of human MSCs in the treatment of sheep with induced ARDS, MSC therapy via intravenous infusion resulted in both significant improvement in hypoxemia and reduced pulmonary edema. In a randomized controlled protocol with low- and high-dose treatment cohorts (5 × 10^6^ vs 10 × 10^6^ MSCs/kg, respectively), the cohort receiving high-dose MSC therapy showed greatest efficacy. No adverse effects from MSC therapy on systemic blood pressure, pulmonary arterial pressure, pulmonary vascular resistance and renal function were observed [33].

Benefits such as those observed by Lee and colleagues in the application of MSC or MSC CM in *ex vivo* analysis of acute lung injury have seen the preclinical exploration of MSC derived EVs as a potential therapy. In an induced ARDS mouse model, Zhu and colleagues studied the benefits of bone marrow-derived MSC EVs against the effect of MSC therapy alone [34]. Reduced lung inflammation and reduced pulmonary edema were observed equally in both groups. In addition to this, EVs have been shown to transfer functional mitochondria to lung epithelial cells similar to that observed in other preclinical MSC trials, resulting in improved ATP production and cell function [29,35].

## Clinical research

Wilson and colleagues have published the results of a clinical Phase I dose-escalation trial on the use of MSCs for the treatment of ARDS. Participants were separated into three dose-dependent cohorts receiving 1 × 10^6^, 5 × 10^6^ and 10 × 10^6^ MSCs/kg, respectively. This early-phase safety trial showed that a single intravenous infusion of allogeneic human MSCs was well tolerated with no MSC related adverse events recorded [36]. An additional early-phase trial by Zheng and colleagues assessing the use of allogeneic adipose-derived MSC therapy at a dose of 1 × 10^6^ MSCs/kg noted that the treatment was safe with no serious adverse events. Serum surfactant protein (a biomarker of lung injury) was significantly lower in the treatment group at day 5 though there was no observed difference in length of hospital stay, ventilator-free days and Intensive Care Unit (ICU) free days at day 28 between the control or treatment groups [37].

In an open label Phase I/II study assessing the benefit of allogeneic bone marrow derived multipotent progenitor cells in the treatment of ARDS, a lowering of 28-day mortality and an increase in both ventilator free and ICU free days was observed [38]. Participants in the treatment cohort received 900 million cells. Treatment was well tolerated with no serious adverse events reported.

The successful use of allogeneic MSC therapy in Influenza induced ARDS has been recently documented with an open-label controlled trial showing significantly lower mortality in the treatment group (17.6%) to the control group (54.5%) [39]. Participants in the treatment group received a dose of 1 × 10^6^ MSCs/kg with up to four infusions. MSC infusions were well tolerated.

Most recently, Leng and colleagues have shown the clinical safety and efficacy of MSC therapy in the treatment of respiratory distress associated with novel coronavirus disease. In this limited pilot trial, ten patients with confirmed COVID-19 infection who had not improved with conventional supportive therapy were enrolled. Post infusion of MSC therapy, participants showed clinical improvement within 2–4 days with resolution of fever and improved oxygen saturation. In the most severely ill patient, C-reactive protein (CRP) was shown to improve from 105.5 to 10.1 g/l after 2 weeks with other biochemical markers of organ impairment improving to normal limits within 2–4 days. This limited trial indicated the potential role in MSC therapy in both the treatment of viral pneumonia and secondary ARDS [40].

## Discussion

ARDS is a condition of acute respiratory failure and associated with a high mortality rate. Current treatments are limited and are primarily supportive. An understanding of the disease pathology involving an unchecked inflammatory cascade and tissue endothelial/epithelial breakdown has led to the investigation of new therapies. MSCs express both immunomodulatory and reparative pathways with both preclinical and initial early-phase clinical trial evidence suggestive of potential therapeutic benefit.

While preclinical trials have shown reproducible benefit from MSC therapy it is important to recognize that mode of delivery (intrapulmonary vs intravenous infusion) differs considerably in these trials. Importantly, preclinical assessment of the biodistribution of MSCs after intravascular infusion has shown predominant localization to the lung [41]. In addition to this, other organs that MSCs are observed to localize to, including the liver, heart and brain, indicate potential additional benefit in management of multiple organ failure which may be associated with ARDS.

In addition to differing modes of application, dosing is inconsistent among animal trials and *in vitro* or *ex vivo* trials do not indicate an appropriate clinical therapeutic dose range. Interestingly, in a preclinical dosing trial assessing the efficacy of MSC therapy in ventilator induced lung injury, Hayes and colleagues demonstrated observed comparable benefits in doses of 2 × 10^6^ MSCs/kg and above [42]. This suggests a dose/response threshold with perhaps limited or no benefit beyond this.

The inconsistency with MSC dosing is also seen in clinical trials with participants receiving a dose range of between 1 and 10 × 10^6^ MSCs/kg and with another single study giving all participants a total of 900 million cells. In addition to this there is consideration to the potential role of multiple infusions with Chen and colleagues showing reproducible benefit and reduced mortality in influenza associated ARDS following multiple infusions of MSC therapy [39].

Despite systematic review of trials involving a total of over 1000 participants receiving intravascular MSC therapy indicating safety with no observed MSC related serious adverse events there remains concern regarding MSC therapy and potential of MSCs to migrate to a site of cancer and promote tumor growth [43]. In contrast, MSCs have also been shown to inhibit growth of some tumors [44,45]. This highlights the importance of structured trials with clear inclusion and exclusion criteria to ensure the appropriate and safe development of MSC therapies.

## Conclusion

Despite improvement in our understanding of the disease pathology of ARDS, it continues to have limited treatment options and poor outcome with a high fatality rate. Preclinical trials assessing MSC therapy have shown promise with observed improvement in biomarkers of inflammation, acute lung injury and improved lung function indicating an anti-inflammatory and reparative process. Early-phase clinical trials have shown safety in the intravenous application of MSC therapy for ARDS with some trials showing initial efficacy indicated by improved ventilator and ICU free days and reduced mortality. MSC therapy represents a promising breakthrough in the active management of a condition, which until now has had limited treatment options.

## Future perspective

As our understanding of the pathology of various diseases improves, regenerative medicine represents an important pathway in future treatment modalities. While many cellular therapies already form part of accepted medical practice (i.e., bone marrow and tissue transplantation), MSCs – with their observed immunomodulatory and reparative mechanisms, hold significant promise. While ongoing research needs to be promoted, initial results in areas with otherwise limited treatment options – including ARDS – are both encouraging and exciting.

**Executive summary**

- Acute respiratory distress syndrome (ARDS) is a condition of acute respiratory failure resulting from noncardiogenic pulmonary edema.
- The pathogenesis of ARDS is understood to be an unregulated inflammatory cascade with both endothelial and epithelial layer damage leading to alveolar fluid collection and pulmonary edema.
- Treatment of ARDS primarily involves supportive care.
- Mortality of ARDS ranges from 25 to 40%.
- Mesenchymal stem cells (MSCs) exert their effect through both immunomodulatory and reparative pathways.
- Preclinical trials have indicated the potential of MSC therapy to reduce mortality, reduce inflammation and limit lung injury.
- Early clinical trials have shown safety in MSC administration for ARDS with promising initial outcome results indicating reduction in inflammatory markers, improvement in lung function and reduced mortality.
- Further, well-controlled research is needed to determine the most effective dose and frequency of MSC therapy in ARDS.

## References

[1] Matthay MA, Ware LB, Zimmerman GA The acute respiratory distress syndrome. J. Clin. Invest. 122(8), 2731–2740 (2012). 22850883 10.1172/JCI60331 PMC3408735

[2] Huppert LA, Matthay MA, Ware LB Pathogenesis of acute respiratory distress syndrome. Semin. Respir. Crit. Care Med. 40(1), 31–39 (2019). 31060086 10.1055/s-0039-1683996 PMC7060969

[3] Rubenfeld GD, Caldwell E, Peabody E Incidence and outcomes of acute lung injury. N. Engl. J. Med. 353(16), 1685–1693 (2005). 16236739 10.1056/NEJMoa050333

[4] Máca J, Jor O, Holub M Past and present ARDS mortality rates: a systematic review. Respir. Care 62(1), 113–122 (2017). 27803355 10.4187/respcare.04716

[5] Tisoncik JR, Korth MJ, Simmons CP Into the eye of the cytokine storm. Microbiol. Mol. Biol. Rev. 76(1), 16–32 (2012). 22390970 10.1128/MMBR.05015-11 PMC3294426

[7] Opitz B, van Laak V, Eitel J, Suttorp N Innate immune recognition in infectious and noninfectious diseases of the lung. Am. J. Respir. Crit. Care Med. 181(12), 1294–1309 (2010). 20167850 10.1164/rccm.200909-1427SO

[8] Imai Y, Kuba K, Neely GG Identification of oxidative stress and Toll-like receptor 4 signaling as a key pathway of acute lung injury. Cell 133(2), 235–249 (2008). 18423196 10.1016/j.cell.2008.02.043 PMC7112336

[9] Corada M, Mariotti M, Thurston G Vascular endothelial–cadherin is an important determinant of microvascular integrity *in vivo* . Pro. Natl Acad. Sci. 96(17), 9815–9820 (1999). 10.1073/pnas.96.17.9815 PMC22293 10449777

[10] Calfee CS, Matthay MA, Kangelaris KN Cigarette smoke exposure and the acute respiratory distress syndrome. Crit. Care Med. 43(9), 1790 (2015). 26010690 10.1097/CCM.0000000000001089 PMC4737582

[11] Moss M, Burnham EL Chronic alcohol abuse, acute respiratory distress syndrome, and multiple organ dysfunction. Crit. Care Med. 31(4), S207–S212 (2003). 12682442 10.1097/01.CCM.0000057845.77458.25

[12] Hibbert K, Rice M, Malhotra A Obesity and ARDS. Chest 142(3), 785–790 (2012). 22948584 10.1378/chest.12-0117 PMC3435141

[13] Caplan AI Why are MSCs therapeutic? New data: new insight. J. Pathol. 217(2), 318–324 (2009). 19023885 10.1002/path.2469 PMC8793150

[14] Caplan AI, Correa D The MSC: an injury drugstore. Cell Stem Cell 9(1), 11–15 (2011). 21726829 10.1016/j.stem.2011.06.008 PMC3144500

[15] Nakagami H, Morishita R, Maeda K, Kikuchi Y, Ogihara T, Kaneda Y Adipose tissue-derived stromal cells as a novel option for regenerative cell therapy. J. Atheroscler. Thromb. 13(2), 77–81 (2006). 16733294 10.5551/jat.13.77

[16] Caplan AI Mesenchymal stem cells. J. Orth. Res. 9(5), 641–650 (1991). 10.1002/jor.1100090504 1870029

[17] Peng L, Jia Z, Yin X Comparative analysis of mesenchymal stem cells from bone marrow, cartilage, and adipose tissue. Stem Cells Dev. 17(4), 761–774 (2008). 18393634 10.1089/scd.2007.0217

[18] Alvarez-Viejo M, Menendez-Menendez Y, Blanco-Gelaz MA Quantifying mesenchymal stem cells in the mononuclear cell fraction of bone marrow samples obtained for cell therapy. Transplant. Proc. 45(1), 434–439 (2013). 23375334 10.1016/j.transproceed.2012.05.091

[19] Baer PC, Geiger H Adipose-derived mesenchymal stromal/stem cells: tissue localization, characterization, and heterogeneity. Stem Cells Int. 2012, (2012). 10.1155/2012/812693 PMC3345279 22577397

[20] Ra JC, Shin IS, Kim SH Safety of intravenous infusion of human adipose tissue-derived mesenchymal stem cells in animals and humans. Stem Cells Dev. 20(8), 1297–1308 (2011). 21303266 10.1089/scd.2010.0466

[21] Izadpanah R, Trygg C, Patel B Biologic properties of mesenchymal stem cells derived from bone marrow and adipose tissue. J. Cell. Biochem. 99(5), 1285–1297 (2006). 16795045 10.1002/jcb.20904 PMC4048742

[22] Kim J, Kang JW, Park JH Biological characterization of long-term cultured human mesenchymal stem cells. Arch. Pharm. Res. 32(1), 117–126 (2009). 19183884 10.1007/s12272-009-1125-1

[23] Wang Y, Han Z, Song Y, Han ZC Safety of mesenchymal stem cells for clinical application. Stem Cells Int. 2012, 652034 (2012). 22685475 10.1155/2012/652034 PMC3363282

[24] Nekanti U Long-term expansion and pluripotent marker array analysis of Wharton's jelly-derived mesenchymal stem cells. Stem Cells Dev. 19, 117–130 (2010). 19619003 10.1089/scd.2009.0177

[25] Lalu MM, McIntyre L, Pugliese C Safety of cell therapy with mesenchymal stromal cells (SafeCell): a systematic review and meta-analysis of clinical trials. PLoS ONE 7(10), e47559 (2012). 23133515 10.1371/journal.pone.0047559 PMC3485008

[26] Ortiz LA, DuTreil M, Fattman C Interleukin 1 receptor antagonist mediates the antiinflammatory and antifibrotic effect of mesenchymal stem cells during lung injury. Proc. Natl Acad Sci. 104(26), 11002–11007 (2007). 17569781 10.1073/pnas.0704421104 PMC1891813

[27] Gupta N, Su X, Popov B, Lee JW, Serikov V, Matthay MA Intrapulmonary delivery of bone marrow-derived mesenchymal stem cells improves survival and attenuates endotoxin-induced acute lung injury in mice. J. Immunol. 179(3), 1855–1863 (2007). 17641052 10.4049/jimmunol.179.3.1855

[28] Lee JW, Fang X, Gupta N, Serikov V, Matthay MA Allogeneic human mesenchymal stem cells for treatment of *E. coli* endotoxin-induced acute lung injury in the *ex vivo* perfused human lung. Proc. Natl Acad. Sci. 106(38), 16357–16362 (2009). 19721001 10.1073/pnas.0907996106 PMC2735560

[29] Islam MN, Das SR, Emin MT Mitochondrial transfer from bone-marrow–derived stromal cells to pulmonary alveoli protects against acute lung injury. Nat. Med. 18(5), 759 (2012). 22504485 10.1038/nm.2736 PMC3727429

[30] Lee JW, Krasnodembskaya A, McKenna DH, Song Y, Abbott J, Matthay MA Therapeutic effects of human mesenchymal stem cells in *ex vivo* human lungs injured with live bacteria. Am. J. Respir. Crit. Care Med. 187(7), 751–760 (2013). 23292883 10.1164/rccm.201206-0990OC PMC3678109

[31] Gupta N, Krasnodembskaya A, Kapetanaki M Mesenchymal stem cells enhance survival and bacterial clearance in murine *Escherichia coli* pneumonia. Thorax 67(6), 533–539 (2012). 22250097 10.1136/thoraxjnl-2011-201176 PMC3358432

[32] Krasnodembskaya A, Samarani G, Song Y Human mesenchymal stem cells reduce mortality and bacteremia in gram-negative sepsis in mice in part by enhancing the phagocytic activity of blood monocytes. Am. J. Physiol. Lung Cell. Mol. Physiol. 302(10), L1003–L1013 (2012). 22427530 10.1152/ajplung.00180.2011 PMC3362255

[33] Asmussen S, Ito H, Traber DL Human mesenchymal stem cells reduce the severity of acute lung injury in a sheep model of bacterial pneumonia. Thorax 69(9), 819–825 (2014). 24891325 10.1136/thoraxjnl-2013-204980 PMC4284068

[34] Yg Zhu, Xm Feng, Abbott J Human mesenchymal stem cell microvesicles for treatment of *Escherichia coli* endotoxin-induced acute lung injury in mice. Stem Cells 32(1), 116–125 (2014). 23939814 10.1002/stem.1504 PMC3947321

[35] Fergie N, Todd N, McClements L, McAuley D, O'Kane C, Krasnodembskaya A Hypercapnic acidosis induces mitochondrial dysfunction and impairs the ability of mesenchymal stem cells to promote distal lung epithelial repair. FASEB J. 33(4), 5585–5598 (2019). 30649987 10.1096/fj.201802056R PMC6436662

[36] Wilson JG, Liu KD, Zhuo H Mesenchymal stem (stromal) cells for treatment of ARDS: a Phase I clinical trial. Lancet Respir. Med. 3(1), 24–32 (2015). 25529339 10.1016/S2213-2600(14)70291-7 PMC4297579

[37] Zheng G, Huang L, Tong H Treatment of acute respiratory distress syndrome with allogeneic adipose-derived mesenchymal stem cells: a randomized, placebo-controlled pilot study. Respir. Res. 15(1), 39 (2014). 24708472 10.1186/1465-9921-15-39 PMC3994204

[38] Bellingan G, Jacono F, Barnard-Smith J Primary analysis of a Phase I/IIstudy to assess MultiStem cell therapy, a regenerative advanced therapy medicinal product (ATMP), in acute respiratory distress syndrome (MUST-ARDS). : American Thoracic Society 2019 International Conference. American Journal of Respiratory and Critical Care Medicine, 201, 7353 (2019).

[39] Chen J, Hu C, Chen L Clinical study of mesenchymal stem cell treating acute respiratory distress syndrome induced by epidemic Influenza A (H7N9) infection, a hint for COVID-19 treatment. Engineering (2020). 10.1016/j.eng.2020.02.006 PMC7102606 32292627

[40] Leng Z, Zhu R, Hou W Transplantation of ACE2-mesenchymal stem cells improves the outcome of patients with COVID-19 pneumonia. Aging Dis. 11(2), 216–228 (2020). 32257537 10.14336/AD.2020.0228 PMC7069465

[41] Toupet K, Maumus M, Peyrafitte J Long term detection of human adipose-derived mesenchymal stem cells after intra-articular injection in SCID mice. Arthritis Rheum. 65, 1786–1794 (2013). 23553439 10.1002/art.37960

[42] Hayes M, Masterson C, Devaney J Therapeutic efficacy of human mesenchymal stromal cells in the repair of established ventilator-induced lung injury in the rat. Anesthesiology 122(2), 363–373 (2015). 25490744 10.1097/ALN.0000000000000545

[43] Ridge SM, Sullivan FJ, Glynn SA Mesenchymal stem cells: key players in cancer progression. Mol. Cancer 16(1), 31 (2017). 28148268 10.1186/s12943-017-0597-8 PMC5286812

[44] Yang L, Zhang Y, Cheng L Mesenchymal stem cells engineered to secrete pigment epithelium-derived factor inhibit tumor metastasis and the formation of malignant ascites in a murine colorectal peritoneal carcinomatosis model. Hum. Gene Ther. 27(3), 267–277 (2016). 26756933 10.1089/hum.2015.135

[45] Chanda D, Isayeva T, Kumar S Therapeutic potential of adult bone marrow–derived mesenchymal stem cells in prostate cancer bone metastasis. Clin. Cancer Res. 15(23), 7175–7185 (2009). 19920103 10.1158/1078-0432.CCR-09-1938 PMC2943933

